# Respiratory syncytial virus infection is associated with an altered innate immunity and a heightened pro-inflammatory response in the lungs of preterm lambs

**DOI:** 10.1186/1465-9921-12-106

**Published:** 2011-08-09

**Authors:** Fatoumata B Sow, Jack M Gallup, Subramaniam Krishnan, Andriani C Patera, JoAnn Suzich, Mark R Ackermann

**Affiliations:** 1 Department of Veterinary Pathology, College of Veterinary Medicine, Iowa State University, Ames, IA 50011, USA; 2 MedImmune, LLC, One MedImmune Way, Gaithersburg, MD 20878, USA

**Keywords:** Lung, neonate, premature infant, immune response, RSV, lamb

## Abstract

**Introduction:**

Factors explaining the greater susceptibility of preterm infants to severe lower respiratory infections with respiratory syncytial virus (RSV) remain poorly understood. Fetal/newborn lambs are increasingly appreciated as a model to study key elements of RSV infection in newborn infants due to similarities in lung alveolar development, immune response, and susceptibility to RSV. Previously, our laboratory demonstrated that preterm lambs had elevated viral antigen and developed more severe lesions compared to full-term lambs at seven days post-infection. Here, we compared the pathogenesis and immunological response to RSV infection in lungs of preterm and full-term lambs.

**Methods:**

Lambs were delivered preterm by Caesarian section or full-term by natural birth, then inoculated with bovine RSV (bRSV) via the intratracheal route. Seven days post-infection, lungs were collected for evaluation of cytokine production, histopathology and cellular infiltration.

**Results:**

Compared to full-term lambs, lungs of preterm lambs had a heightened pro-inflammatory response after infection, with significantly increased MCP-1, MIP-1α, IFN-γ, TNF-α and PD-L1 mRNA. RSV infection in the preterm lung was characterized by increased epithelial thickening and periodic acid-Schiff staining, indicative of glycogen retention. Nitric oxide levels were decreased in lungs of infected preterm lambs compared to full-term lambs, indicating alternative macrophage activation. Although infection induced significant neutrophil recruitment into the lungs of preterm lambs, neutrophils produced less myeloperoxidase than those of full-term lambs, suggesting decreased functional activation.

**Conclusions:**

Taken together, our data suggest that increased RSV load and inadequate immune response may contribute to the enhanced disease severity observed in the lungs of preterm lambs.

## Introduction

Respiratory syncytial virus (RSV) is the most common cause of severe lower respiratory tract disease in children, with the highest morbidity occurring in the youngest infants [1-3]. Preterm infants are particularly susceptible to the disease, due in part, to their immature immune system, low birth weight, and lung immaturity. Recent literature suggests that infants born 33-35 weeks of gestation are at similar risk for RSV-related lower respiratory tract infection as infants born less than 32 weeks of gestation (reviewed in [4]). RSV-related hospitalization rates of infants born at 33-35 weeks of gestation are twice as high as those of term infants [5]. Currently, there is no RSV vaccine, and the only preventive treatment for the disease involves passive immunization with antibody against RSV [3,6].

The pathogenesis of RSV disease is not well understood; however, a multifactorial process that includes virus-induced pathology and a concomitant exaggerated host immune response may play critical roles in the disease process [7,8]. Increased viral load is thought to contribute to the severity of disease [9], although the underlying mechanisms responsible for increased viral replication in some individuals are not completely understood. RSV specific immunity is also believed to be involved in the mechanism of severe lower respiratory tract disease, based on experiences with a formalin-inactivated RSV vaccine preparation during which vaccinated infants developed an enhanced eosinophilia to re- infection [10,11].

Previously, our laboratory has shown that preterm lambs infected with bovine RSV (bRSV) developed clinical symptoms and lesions that paralleled the course of disease observed in human infants suffering from severe RSV disease [12]. Moreover, they had a reduced capacity for RSV antigen clearance compared to full-term lambs [12]. The precise mechanisms underlying the age-dependent disease severity and the lack of RSV clearance were not investigated. Therefore, we studied the relationship between developmental age and immunological competence in response to RSV infection by assessing for differences in the nature of the inflammatory response in the lungs of preterm and full-term lambs. We hypothesized that the increased viral load in preterm lambs is associated with an altered distribution and functional activation of immune cells and increased lung pathology that differs from full-term lambs.

## Materials and methods

### Animals

Animal use and experimental procedures were approved by Iowa State University's Animal Care and Use Committee. We used banked lung samples from lambs that were used in another study to characterize bRSV infection in the lamb model [12]. Briefly, lambs were delivered preterm by Caesarian section at 138 days of gestation (term is 147 days) or full-term by natural birth. Both groups were inoculated with bRSV strain 375 (20 ml at 10^3-4 ^TCID_50_/ml) or sterile cell growth media (20 ml) via the intratracheal route. Lambs (n = 6 for the preterm control group, n = 6 preterm RSV, n = 3 full-term control, n = 5 full-term RSV) were euthanized with sodium pentobarbital i.v. 7 days post-infection. Sections of the right middle lung lobe were removed, snap-frozen in liquid nitrogen and stored at -80°C for real-time RT-PCR and other functional assays. Additional sections of the right cranial lung lobe were fixed in 10% neutral-buffered formalin for histopathology and immunohistochemical analyses.

### Histopathology, lesions and staining scores

After formalin fixation, lung samples were embedded in paraffin blocks. Lung sections (5 μm) were stained with H&E to assess histologic changes, including cellular infiltration and epithelial alterations. Cellular infiltration and epithelial hyperplasia were described and scored by a pathologist (MRA) using a minimum of three 20X fields in each lung section.

The extent of neutrophil infiltration in bronchioles and alveoli surrounding affected bronchioles in the 20X fields were scored with the following scale: 0 = no significant neutrophil infiltration, 1 = detectable presence of 1 to 5 neutrophils within bronchioles/alveolar lumens in 20X field, 2 = groups (greater than 5) of cells in bronchioles/alveolar lumens in 20X field, 3 = accumulation of cells that fills bronchioles/alveolar lumens in 20X field, 4 = dense infiltration of cells that distends bronchiolar and alveolar lumens in a 20X field. Macrophage infiltration was determined using the same scoring system.

Lymphocytes and plasma cell infiltration into the alveolar and bronchiolar institium were scored with the following scale of 20X fields: 0 = no significant infiltration, 1 = presence of infiltrating cells in alveolar septa or bronchiolar interstitium, 2 = accumulation of several cells within alveolar septa or bronchiolar interstitium, 3 = accumulation of cells that expands the alveolar septa or bronchiolar interstitium, 4 = accumulation of cells that forms dense aggregates in the alveolar septum or bronchiolar interstitium.

With delivery of RSV inoculum in this study, lesions were present within distal bronchioles and alveoli [12]. In contrast, bronchi and large proximal bronchioles lack lesions and RSV antigen. Thus only distal bronchioles were scored for epithelial alterations and these airways were relatively uniform in size and diameter. Bronchiolar epithelial hyperplasia was scored with the following scale: 0 = no lesion, 1 = first detectable thickening resulting in mild hypertrophy of bronchiolar cells and occasional cells more than one layer thick, 2 = thickening that is double the normal thickness of a single cuboidal cell, 3 = thickening that protrudes into and partially occludes the airway lumen and slight expansion/distension of the airway, 4 = thickening that occludes the airway lumen resulting in a very narrow (slit) lumen and marked distension of the airway.

Lung sections were also stained with periodic acid-Schiff (PAS) to assess the stage of epithelial differentiation/maturity as a measure of glycogen retention in type II pneumocytes. The thickened epithelial layers had variable levels of staining avidity, and ten alveoli were scored per lung section from each lamb. Alveoli were similar in size which ranged between 100-200 μm in diameter. Scoring was based on predetermined scale: 0, no detectable stain; 1, faint but detectable stain; 2, cytoplasmic staining present in less than 30% of alveolar epithelial cells; 3, cytoplasmic staining present in more than 60% of alveolar epithelial cells.

### RNA isolation and one-step real-time reverse transcriptase qPCR

Total RNA was isolated from lung tissue as previously described [13] using a procedure based on TRIzol reagent (Invitrogen, Carlsbad, CA). The RNA samples were DNase-treated using TURBO DNase (TURBO DNA-free kit, Ambion, Austin, TX). RNA concentrations and purity were measured by absorbance readings at 260 nm and 280 nm.

Initial RT-qPCR analysis involved running a test plate for each target to identify the best RNA dilution ranges in which PCR inhibition was not observed, and where amplification efficiencies were better than 80%. Test-plate set-up parameters and analysis were performed using PREXCEL-Q [14]. RNA samples were used at 0.784 ng/μl in fluorogenic one-step real-time qPCR reactions. Each 20 μl reaction contained 4.8 μl of RNA sample, 775 nM primers, 150 nM TaqMan hydrolysis probe (5'-6FAM, 3'-TAMRA-quenched or 5'-6FAM, 3'-MGBNFQ), nuclease-free water, 5.5 mM MgSO_4_, SuperScript III RT/Platinum Taq mix (Invitrogen), and One-step reaction mix with ROX (Invitrogen). Primers and probes sequences for all targets except perforin can be found in [13,15]. Sequences for perforin are: Fwd: 5'-GCCGTGAGTAAGTATGTGACTGACA; Rev: 5'-GCCAGGACACATGCATTGG; Probe: 6FAM-TGGAGGGACTGCAACC-MGBNFQ). The samples were placed in duplicate wells of 96-well plates (Eppendorf, Westbury, NY). No-template control (NTC) and no-reverse transcriptase (NRC) reactions consistently demonstrated lack of target contamination and negligible genomic DNA contamination. The GeneAmp 5700 Sequence Detection System (Applied Biosystems) was programmed for thermocycling at 15 min 55°C, 2 min 95°C, and 50 cycles of 15 s 95°C, 30 s 58°C. Quantification cycle (Cq) values generated by the GeneAmp 5700 system were further assessed using custom Excel files employing the efficiency-corrected standard curve-based relative quantification (Pfaffl) approach for qPCR [16]. Results are expressed as gene (mRNA) expression of infected samples relative to that of uninfected samples.

### Immunohistochemistry (IHC) and scoring system for myeloperoxidase and caspase 3

Sections were made from paraffin-embedded blocks on the same day and were stained for either myeloperoxidase or caspase 3 in a single batch for uniformity. Briefly, paraffin-embedded lung sections were deparaffinized, blocked in 10% normal goat serum + 10% normal swine serum in PBS, then stained with 1:300 rabbit anti-human myeloperoxidase (Dako, Carpinteria, CA) or 1:1000 rabbit anti-human/mouse active caspase 3 (R&D Systems, Minneapolis, MN) followed by 1:300 biotinylated goat anti-rabbit IgG (Kirkegaard & Perry Laboratories, Gaithersburg, MD), streptavidin-conjugated horseradish peroxidase (BioGenex) and Nova Red (Vector, Burlingame, CA). Finally, the slides were counterstained with Harris' hematoxylin. Scoring for myeloperoxidase (MPO) were made in 40 × sections from at least 3 fields per lung lobe per animal where 0 = no staining, 1 = 1-10% of neutrophils had MPO immunoreactivity (IR; MPO-IR), 2 = 11-30% of neutrophils had MPO-IR; 3 = 30-60% of neutrophils had MPO-IR and 4 = > 60% of neutrophils had MPO-IR. Scores for caspase 3 IR ranged from 0-4 and were assigned to bronchiolar and alveolar areas. In bronchioles: (0 = no staining, 1 = 1-10%, 2 = 11-30%, 3 = 30-60%, 4 = more than 60% of airway epithelia had immunoreactivity to caspase 3). In alveoli, staining occurred in consolidated areas, containing lymphocytes, plasma cells, macrophages and alveolar epithelia: (0 = no staining, 1 = 1-10%, 2 = 11-30%, 3 = 30-60%, 4 = more than 60% of consolidated areas had immunoreactivity to caspase 3).

### Lung nitrite levels

Lungs were homogenized in a Tissue Protein Extraction Reagent (T-PER; Fisher Science, Hanover Park, IL) at a ratio of 1 g tissue/20 ml T-PER. After the addition of protease inhibitors cocktail tablets (Roche), the samples were homogenized using an Omni TH homogenizer (Omni International). Protein concentration was determined using the Coomassie Plus Protein Assay Reagent (Fisher). Nitrite levels in lung homogenates were measured using a commercially available Griess reaction assay kit as per the manufacturer's instructions (Promega, Madison, WI). The optical density at 550 nm (OD_550_) was measured using a microplate reader. Nitrite concentrations were calculated by comparison with the OD_550 _of standard solutions of sodium nitrite.

### Lung arginase activity

Arginase levels in lung homogenates were measured using a commercially available assay (QuantiChrom arginase assay kit by BioAssay System, Hayward, CA). Methods followed the manufacturer's recommendations. Arginase activity (units/L) in all lung samples was calculated by comparison with the OD_520 _of standard solution of urea.

### Statistical analysis

The results were analyzed by one-way ANOVA using SAS version 9.1 (SAS Institute, Cary, NC). Mean values of relative mRNA expression levels of control animals and RSV-infected animals were compared at *p < 0.05, **p < 0.01, and ***p < 0.001 and expressed as means ± SE.

## Results

### RSV infection induces higher relative mRNA levels of innate immune mediators in preterm lambs than full-term lambs

**Figure 1:**
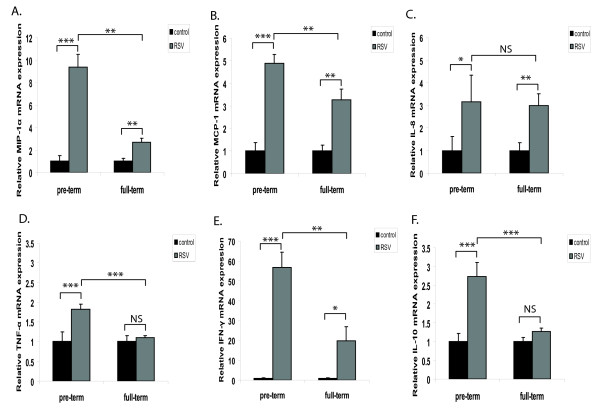
**RSV infection induces higher levels of innate immune mediators in preterm lambs than full-term lambs**. mRNA levels were measured by qPCR and expressed as fold induction relative to control animals at the same time point. Preliminary correlation analysis of qPCR data determined that the level of each mediator was similar between control animals of either age groups. (A) MIP-1α, (B) MCP-1, (C) IL-8, (D) TNF-α, (E) IFN-γ and (F) IL-10. The data represent the mean ± SE. *p < 0.05, **p < 0.01, ***p < 0.001 by ANOVA. NS, p > 0.05.

Previously, our laboratory demonstrated that the bRSV challenge dose used to infect lambs caused clinical signs and pulmonary lesions typical of severe human RSV disease [12]. Moreover, we reported that preterm lambs had enhanced viral load in their lungs compared to full-term lambs [12]. In order to compare the immune response during RSV infection across age groups in lambs, we measured the expression of several key innate immunity mediators at the mRNA level. Levels of MIP-1α, MCP-1, IL-8, TNF-α, IFN-γ and IL-10 (Figure 1A-F) were significantly increased in RSV-infected animals compared to control animals of the same age group. Full-term lambs infected with RSV had statistically significant enhanced expression of MIP-1α, MCP-1, IL-8 and IFN-γ, but no significant changes in the expression of TNF-α and IL-10 when compared to control animals of the same age group. Comparisons across age groups following infection revealed that expression of MIP-1α, MCP-1, IL-10, TNF-α and IFN-γ were significantly increased by infection in preterm lambs compared to full-term lambs. These results suggest that an inadequately enhanced immune response occurs in the lungs of preterm lambs after RSV infection.

### RSV infection is associated with airway structural changes and increased caspase 3 staining in preterm lambs

**Figure 2:**
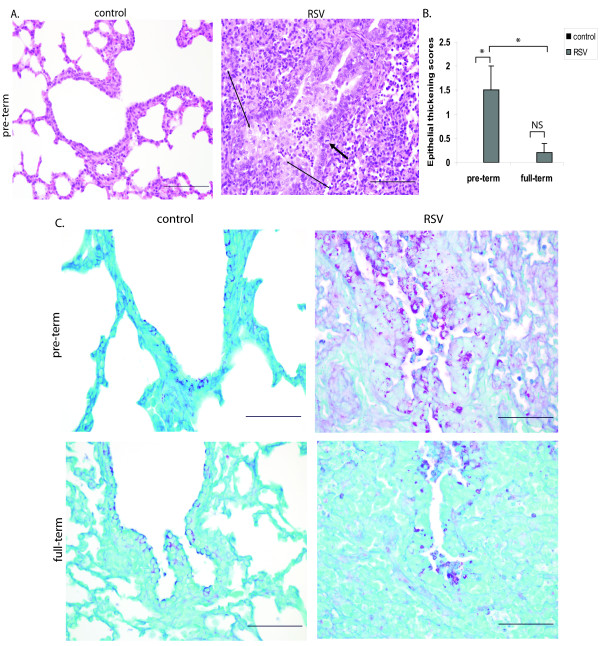
**RSV infection is associated with airway structural changes in the preterm lung**. (A) Representative H&E stain of lung sections from control preterm lamb and RSV-infected preterm lamb. Long arrows represent epithelial thickening, and the short arrow represents a mitotic figure. Bars = 200 μm. (B) Lung sections from each animal were stained with H&E, and epithelial thickening (epithelial cell proliferation/hyperplasia) was scored (see materials and methods section for scoring system). (C) Representative Periodic Acid Schiff (PAS) stain of lung sections from preterm and full-term lambs comparing control to RSV-infected groups. Lung sections were examined under a 20X field. Bars = 200 μm. The data represent the mean ± SE. *p < 0.05 by ANOVA. NS, p > 0.05.

During infection, epithelial cell injury occurs and is associated with proliferation of nearby epithelia to replace injured cells (epithelial cell thickening/hyperplasia). Consistent with this, we determined that control lambs of either age group lacked lesions and areas of epithelial hyperplasia at 7 days post-inoculation with medium. However, 7 days post RSV infection, variable degrees of thickening of the epithelial lining were present in multifocal bronchioles of preterm (long arrows in Figure 2A), and to a lesser degree, full-term lambs (Figure 2B). This was due to hyperplasia of the bronchiolar epithelium with the presence of occasional mitotic figures (short arrow; Figure 2A) and formation of occasional syncytial cells (data not shown). The epithelial cells were cuboidal to polygonal and formed one to several layers in thickness. Epithelial cells in the areas of extensive epithelial thickening were associated with intense staining for PAS (Figure 2C), indicating increased glycogen retention and cellular immaturity. Lungs of RSV-infected preterm lambs had significantly more PAS^+ ^cells than those of RSV-infected full-term lambs (Figure 2C). However, a decrease in epithelial PAS staining was observed during maturation of lung, independent of infection (Figure 2C). This finding, which is consistent with published data comparing the distribution of PAS^+ ^cells through ontogeny in the ovine lung [17], suggests that preterm birth and RSV infection may be independent factors that contribute to the increased epithelial glycogen retention.

**Figure 3:**
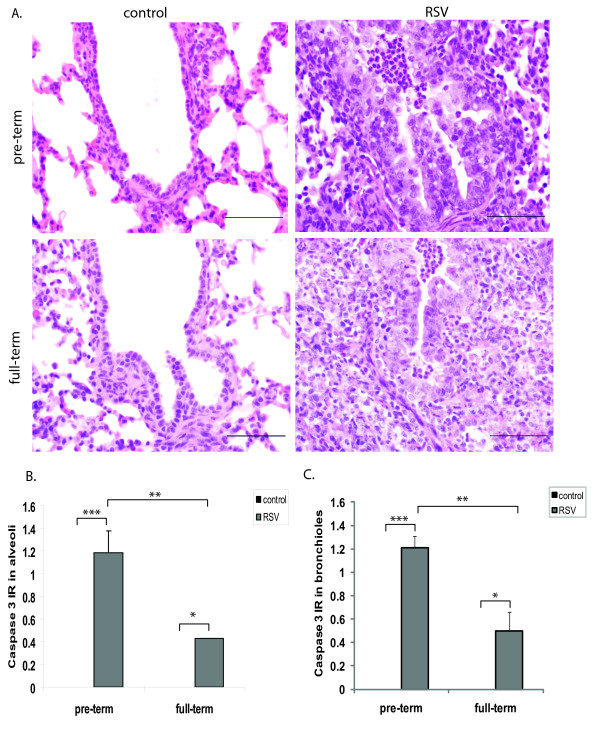
**RSV infection is associated with lung pathology and enhanced caspase 3 staining in the preterm lung**. (A) Representative Haematoxylin and Eosin (H&E) stain of lung sections from preterm and full-term lambs comparing control to RSV-infected groups. Lung sections were examined under a 20X field. Bars = 200 μm. Immunoreactivity (IR) to caspase 3 in alveoli (B) and bronchioles (C) was scored in lungs of control and RSV-infected animals. The data represent the mean ± SE. *p < 0.05, **p < 0.01, ***p < 0.001 by ANOVA.

In addition to the epithelial hyperplasia, pulmonary microscopic lesions were present in RSV-infected animals (Figure 3A). These lesions were similar in both preterm and full-term lambs, but were often accentuated in the preterm lambs. Briefly, multifocal bronchioles of all lambs contained variable infiltrates of neutrophils, occasional degenerate epithelial cells and cell debris with occasional macrophages. Alveolar lumens around these bronchioles had increased numbers of alveolar macrophages with cell debris and small amounts of seroproteinaceous fluid. Infiltrates of lymphocytes and plasma cells were present in alveolar septa and around bronchioles in the bronchiolar adventitia and, to a lesser degree, in the bronchiolar lamina propria (Figure 3A). The observed lung pathology is consistent with RSV infection and has been similarly described in RSV-infected humans [18], cattle (reviewed in [19]) and lambs [12,20-22].

To assess the apoptotic response, lung caspase 3 expression was detected by immunohistochemistry (IHC). Caspase 3 immunoreactivity (IR) was seen in alveolar areas of both groups of infected lambs. However, alveoli of RSV-infected preterm lambs had significantly enhanced IR to caspase 3 when compared to full-term lambs (Figure 3B). A similar trend and level of caspase 3 IR was also observed in bronchioles of infected lambs (Figure 3C). The enhanced viral load observed in lungs of preterm lambs may contribute to the pro-apoptotic response observed.

### Neutrophils of RSV-infected preterm lambs have reduced myeloperoxidase levels compared to full-term lambs

**Figure 4:**
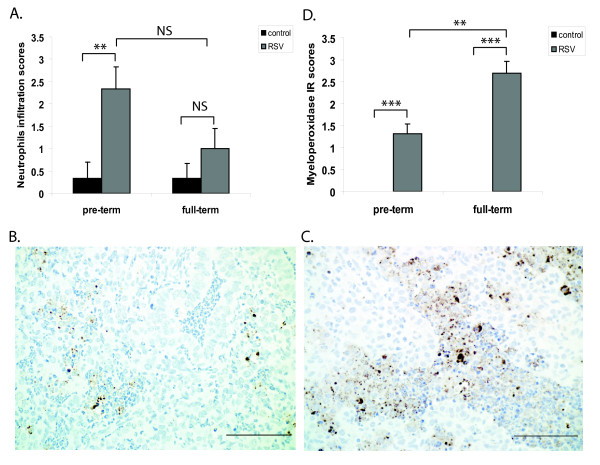
**Neutrophils of RSV-infected preterm lambs have reduced myeloperoxidase levels compared to full-term lambs**. (A) Scoring of neutrophil infiltration in the lung sections of each animal. Histochemical analysis of myeloperoxidase expression in lungs of (B) RSV-infected preterm lambs and (C) RSV-infected full-term lambs. (D) Scores were assigned in lungs of control and RSV-infected animals based on IR to myeloperoxidase. The data represent the mean ± SE. **p < 0.01, ***p < 0.001 by ANOVA. NS, p > 0.05. Bars = 100 μm.

The primary RSV infection in infants is characterized by a strong neutrophil influx into the airways [23-25]. Increased numbers of neutrophils were also detected in lungs of preterm and full-term RSV-infected lambs compared to control lambs (Figure 4A). We also assessed the levels of myeloperoxidase, a key constituent of neutrophils cytotoxic armament [26], by IHC in lung sections. RSV infection was associated with myeloperoxidase IR in both preterm (Figure 4B) and full-term (Figure 4C) lambs when compared to control lambs of the same age groups. However, comparisons across age groups revealed that myeloperoxidase IR was significantly reduced in preterm lambs infected with RSV compared to full-term lambs infected with RSV (Figure 4D). MPO levels in control lambs of either age group were minimal to non-detectable because there were few neutrophils in lung sections from those animals. As neutrophils from myeloperoxidase-deficient individuals kill microorganisms poorly [27,28], our results suggest that neutrophils from RSV-infected preterm lambs may have reduced functionality compared to RSV-infected full-term lambs.

### Macrophages from preterm lambs are recruited to the lung after infection, but produce less nitric oxide than infected full-term lambs

**Figure 5:**
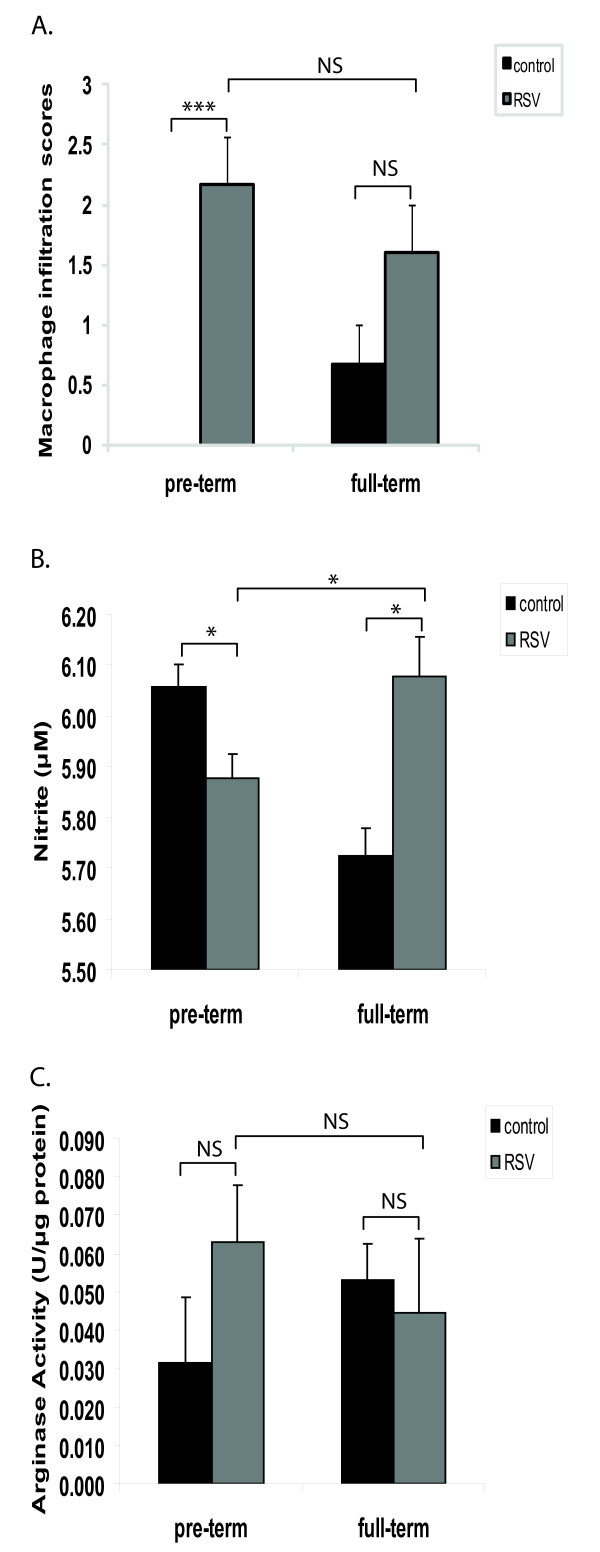
**Macrophages from preterm lambs are recruited to the lung but are differentially activated after infection**. (A) Scoring of macrophage infiltration in the lung sections of each animal. (B) Nitrite levels were measured in whole lung homogenates using the Griess reaction. (C) Arginase activity was measured in lung homogenates and expressed as units per μg of protein sample. The data represent the mean ± SE. *p < 0.05, ***p < 0.001 by ANOVA. NS, p > 0.05.

Because preterm lambs have increased viral loads in the lung [12], we hypothesized that antigen presenting cells, in particular macrophages, were not effectively recruited to the lungs. However, histological analysis of lung sections from preterm lambs in this study indicated that macrophage infiltration was significantly increased by infection when compared to uninfected animals (Figure 5A). In full-term lambs the differences in macrophage numbers between control and infected lungs were not statistically significant (Figure 5A). These findings suggest that the inability of preterm lambs to clear virus from their lungs was not dependent on macrophage numbers.

Since macrophage numbers were similar between preterm and full-term lambs, we reasoned that the functional activity of macrophages of preterm lambs might differ. Whereas the generation of nitric oxide is a hallmark of classically activated macrophages, the phenotype of alternatively activated macrophages includes induction of arginase 1 and reduced levels of nitrate (reviewed in [29]). We measured levels of nitrite, a stable breakdown product of nitric oxide, and arginase in lung homogenates from both groups of lambs. While full-term animals displayed an increase in nitrite levels in their lungs following infection, those of preterm animals were significantly reduced by infection (Figure 5B). No significant changes in arginase levels were measured in either group of lambs regardless of RSV infection (Figure 5C). These findings suggest that macrophages are recruited following RSV infection, but have an altered functional state in the preterm lung.

### Lungs from infected preterm lambs display similar trends in lymphocytic infiltration and functional state compared to full-term lambs

**Figure 6:**
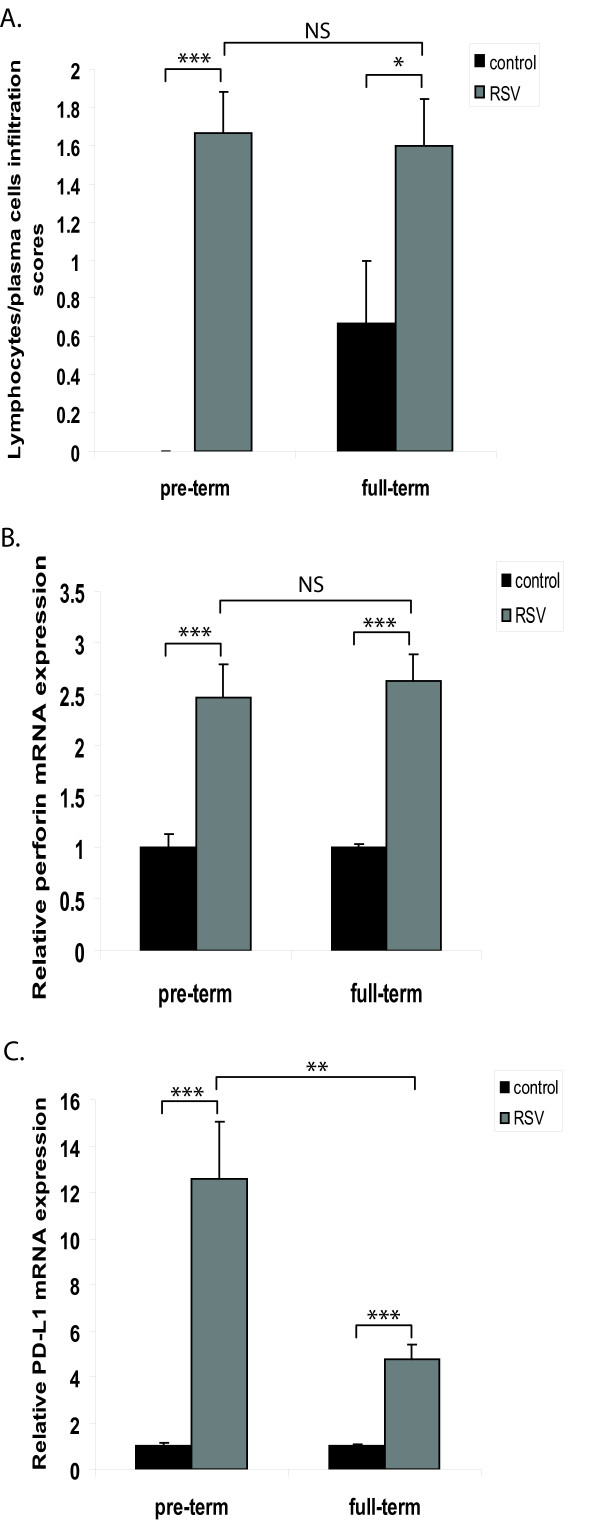
**Lungs from infected preterm lambs display similar trends in lymphocytic infiltration compared to full-term lambs**. (A) Scoring of lymphocytes and plasma cells infiltration in the lung sections of each animal. (B) Perforin mRNA levels were measured by qPCR and expressed as fold induction relative to control animals of the same time point. (C) PD-L1 mRNA levels were measured by qPCR and expressed as fold induction relative to control animals of the same time point. For qPCR results, preliminary correlation analysis of qPCR data determined that the level of each mediator was similar between control animals of either age groups. The data represent the mean ± SE. *p < 0.05, **p < 0.01, ***p < 0.001 by ANOVA. NS, p > 0.05.

Scoring for lymphocytes and plasma cell infiltration revealed that RSV infection induced comparable cellular recruitment in the lung in both preterm and full-term lambs (Figure 6A). The functional state of the lymphocytes was assessed through perforin mRNA (Figure 6B), whose levels were induced by RSV. Similar to the cellular infiltration, perforin mRNA was induced at comparable levels regardless of the age of the infected animal (Figure 6B). The expression of PD-L1, a negative regulator of T cell function was also measured: RSV infection significantly induced PD-L1 mRNA expression in both groups of lambs. However, a comparison between age groups showed that PD-L1 levels in RSV-infected preterm lambs were significantly higher than in RSV-infected full-term lambs (Figure 6C). Taken together, these results suggest that lymphocytic function may be further reduced in preterm lambs than full-term lambs.

## Discussion

Given that infants born prematurely are at higher risks of severe lower respiratory infections with RSV [30,31], we sought to identify potential factors responsible for the age-dependent susceptibility to this pathogen. In this study, we extended characterization of our preterm lamb RSV-infection model [12] by more thoroughly evaluating the immunological aspects of the disease. Herein, we have provided data indicating that during RSV infection, preterm lambs have a heightened innate immune response associated with significant airway structural changes and defects in the distribution and activation of immune cells crucial to host defense against RSV. These responses were less pronounced in full-term lambs. The inappropriate immune response observed in preterm lambs could be attributed to the enhanced viral load in the lungs or to the role for developmental age in mounting an appropriate anti-RSV response. Correlation analysis of the qPCR data showed that the expression of all targets assessed in this study did not differ in lungs of either uninfected preterm and full-term lambs (data not shown).

The recognition of RSV by pathogen-associated molecular patterns by PRRs such as TLRs and RIG-1 [32] results in a cascade of signaling events mediated by NF-κB. During an infection with RSV, these signaling events lead to the expression of cytokines and chemokines, which mediate pro-inflammatory functions to recruit and activate cells, but also regulate the pro-inflammatory state (reviewed in [33,34]). In the current study, RSV-infected preterm lambs had a heightened pro-inflammatory response in their lungs compared to full-term lambs. The observed heightened levels of MIP-1α, MCP-1, IFN-γ, TNF-α and IL-10 in RSV-infected preterm lambs may be due to the increased viral load observed in that group of animals, or to age-related inappropriate transcriptional regulation that contributes to increased pathology, reduced viral clearance, and enhanced disease severity.

RSV predominantly infects airway epithelial cells, which form the first line of defense against the virus and are the site of disease-associated inflammation. RSV infection of preterm lambs was associated with changes in the bronchiolar epithelium, characterized by significant epithelial thickening and increased presence of PAS staining in epithelial cells. At present, it is unclear whether the epithelial thickening and the increased PAS staining detected in the preterm lambs are due to increased RSV replication, to enhanced levels of immune mediators in the lambs, or a combination of both. It is likely that the epithelial thickening, the intra-airway inflammation and bronchoconstriction due to inflammation partially obstruct the bronchiolar lumen and impairs airflow in the preterm lung. Such changes would contribute to impaired gaseous exchange. Additional studies on physiological measurements of airflow and gas exchange would provide important information on the disease process.

Cellular mediators of inflammation, including neutrophils, macrophages, T cells, B cells and eosinophils play key roles in RSV disease in humans (reviewed in [35]). Our findings suggest that these mediators are also involved in the immune response triggered by RSV in lambs. Primary RSV infection in infants is characterized by a strong neutrophil influx [23-25]. In lambs, the neutrophil influx in the airways suggests that these cells might be important contributors to RSV disease. Reports in the literature describing the neutrophil defense system in humans at birth have determined that the neutrophil response is immature and functionally suboptimal [36,37]. Our current study is consistent with these reports in that, despite similar neutrophil infiltration at the site of infection, neutrophils from preterm lambs had significantly lower myeloperoxidase levels than full-term lambs following RSV infection. In addition, the ratio of myeloperoxidase IR to neutrophil infiltration scores (0.56 in preterm lambs, compared to 2.7 in full-term lambs) suggests that the levels of myeloperoxidase per neutrophil is greater in the full-term lambs.. In accordance with our findings, myeloperoxidase levels are significantly reduced in infants born prematurely, but not in term neonates (reviewed in [38]). In addition to prematurity, it is likely that the inappropriate immune response, concomitant with the enhanced viral load in the lung, play a role in modulating myeloperoxidase levels in neutrophils in preterm lambs. To our knowledge, this is the first study to report the role of RSV infection on myeloperoxidase levels during primary infection in preterm lambs.

Although macrophages were recruited to the lungs of both preterm and full-term lambs following RSV infection, the decreased nitrite levels and the trend towards increased arginase activity in lungs of infected preterm lambs suggest that macrophages are either immature or differentially activated in the preterm lung infected with RSV. Alternatively activated macrophages also fail to produce nitric oxide through the induction of arginase and are unable to efficiently kill intracellular pathogens (reviewed in [39]). Based on nitrite levels and arginase activity, our data indicates that macrophages in the RSV-infected preterm lung may have a sub-optimal activation state, resulting in an ineffective immune response to viral infection and poor viral clearance. In addition, our finding that RSV-infected preterm lambs have enhanced caspase 3 IR is in agreement with a study of infants' lungs with fatal lower respiratory tract illness caused by RSV [40]. In the preterm lung, these apoptotic cells are probably not cleared by the macrophages, thus contributing to an inappropriate immune response for viral clearance.

RSV infection can dysregulate RSV-specific cytolytic T cells expression and suppress memory development in the respiratory tract. A rapid loss of RSV-specific memory CD8^+ ^cells in the lungs after infection has been noted in mice (reviewed in [41]). In addition, RSV has been reported to inhibit the development of cytotoxic responses *in vivo*, by suppressing CD4 lymphocyte function [42], interfering with antigen-dependent T cell receptor signaling [43], or inducing lymphocyte apoptosis [44] among other mechanisms. Although we observed no significant differences in lymphocytic infiltration or in the expression levels of components of the lymphocyte lytic machinery (perforin/granzyme) in this study, RSV infection significantly induced the expression levels of PD-1 (data not shown) and PD-L1 in the ovine lung. Because the PD-1/PD-L1 pathway plays an important role in regulating T cell exhaustion [45-48], it is conceivable that other functional aspects of CD8^+ ^cells, such as their capacity to degranulate, are impaired in the RSV-infected ovine lung.

There appears to be a correlation between degrees of cellular infiltration and chemokine levels between preterm and full-term lambs. Similar levels of neutrophils are present in the infected lungs of both preterm and full-term lambs, which correlate with similar levels of IL-8 in the two groups of infected lambs. In the absence of infection, lungs of lambs of either age group have little to no macrophage infiltration. However, in response to RSV infection, the relative increase above baseline in lung macrophage numbers appears to be higher in preterm lambs (infiltration scores of 0 to 2.17 in preterm lambs compared to infiltration scores of 0.67 to 1.6 in full-term-lambs). This is consistent with overall higher levels of MCP-1 and MIP-1α following infection in preterm lungs and a relatively greater elevation in these mediators compared with uninfected baseline levels (for MCP-1: 4.88-fold increase in preterm compared to 3.26-fold in full-term; for MIP-1α: 9.4-fold increase in preterm compared to 2.7-fold in full-term). These observations suggest that a heightened immune response to RSV infection may occur in preterm lambs to compensate for an initial deficit in baseline levels in the immature non-infected lung. This initial deficit, together with the increased viral load, the overall exaggerated response and altered activation state of the cells may contribute to the increased pathology observed.

## Conclusions

In summary, the current study demonstrated that RSV infection of preterm lambs resulted in enhanced levels of pro-inflammatory mediators, including cytokines and chemokines. Lungs of RSV-infected preterm lambs, but not full-term lambs, had marked structural changes, as evidenced by epithelial thickening, increased PAS staining and increased caspase 3 staining in bronchioles and alveoli. Although the expression of caspase 3 suggests the activation of the apoptotic machinery as a result of viral infection of cells, the inefficient innate immune responses mediated in part by the phenotypically altered neutrophils and macrophages likely contribute to the enhanced disease pathology and severity. The similarities in the severity of RSV between lambs and humans in regards to viral susceptibility, clinical responses, lesions and immune responses highlight the utility of the pre-term lamb model to study pathogenesis of RSV. In addition, the ability to induce premature birth in lambs provides an excellent opportunity to study the mechanisms underlying the age-dependent susceptibility of human infants to RSV. Finally, the outbred nature of lambs is a useful characteristic to determine the genetic basis for susceptibility to, and re-infections with RSV.

## Abbreviations used in this paper

IFN: interferon; IHC: immunohistochemistry; IL-: interleukin; IR: immunoreactivity; MCP: monocyte chemotactic protein (also called CCL2); MIP1-α (also called CCL3); PD-L1: programmed death ligand 1; RT-qPCR: reverse transcriptase quantitative polymerase chain reaction; RSV: respiratory syncytial virus; TNF-α: tumor necrosis factor α.

## Competing interests

This work was supported in part by MedImmune, LLC. SK, ACP and JS are employees of MedImmune, LLC. FBS is supported by MedImmune, LLC.

## Authors' contributions

FBS designed, performed the immunological experiments and data analysis for this study, and wrote the manuscript. JMG assisted in the design, performance and analysis of the real-time RT-PCR experiments and edited the manuscript. SK, ACP, and JS provided intellectual input and editorial assistance for the study, participated in the writing and editing of the manuscript, and provided funding for the project. MRA was the principal investigator who oversaw the project, performed the necropsy/tissue collection/lung tissue scoring, participated in the writing and editing of the manuscript, and provided intellectual input and funding for the project. All authors read and approved the final manuscript.
